# Dihydroquercetin and Related Flavonoids in Antioxidant Formulations with α-Tocopherol

**DOI:** 10.3390/ijms26125659

**Published:** 2025-06-13

**Authors:** Vera Olicheva, Vladimir Beloborodov, Shamimeh Sharifi, Anna Dubrovskaya, Anastasiya Zhevlakova, Irina Selivanova, Igor Ilyasov

**Affiliations:** 1 Department of Chemistry, Nelyubin Institute of Pharmacy, Sechenov First Moscow State Medical University, Trubetskaya Street 8/2, 119048 Moscow, Russia; olicheva_v_v@student.sechenov.ru (V.O.); vlbe@list.ru (V.B.); shamimsharifi1376@gmail.com (S.S.); anna.mma@list.ru (A.D.); zhevlakova_a_k@staff.sechenov.ru (A.Z.); selivanova_i_a@staff.sechenov.ru (I.S.); 2 Faculty of Fundamental Medicine, Lomonosov Moscow State University, 27/1 Lomonosovsky Avenue, 119991 Moscow, Russia

**Keywords:** dihydroquercetin, tocopherol, antioxidant formulation, flavonoid, synergy, additive, ABTS, mixture effect, hydrophilic antioxidants, lipophilic antioxidants

## Abstract

The concomitant utilization of flavonoids and α-tocopherol has the potential to establish a comprehensive antioxidant system that operates in both hydrophilic and lipophilic environments. The objective of this study was to examine the antioxidant interactions between dihydroquercetin, a flavonoid that has shown promise in various studies, as well as structurally related flavonoids, and α-tocopherol in various ratios. The antioxidant capacity was assessed using two ABTS^•+^ radical-cation inhibition assays: a decolorization assay and a lag-time assay. The results of this study indicated that formulations of dihydroquercetin, quercetin, rutin, or morin with α-tocopherol exhibited additive or mildly subadditive interactions, independent of their ratios. A two-phase pattern was exhibited by the lag-time data, which, in comparison with individual components, allowed us to suggest that α-tocopherol appeared to dominate the initial phase of radical scavenging, while flavonoids became active in the later phase. This finding indicates that α-tocopherol may play a role in protecting flavonoids from premature oxidation in alcoholic media. The findings could prove valuable for the rational design of antioxidant formulations in the nutraceutical, cosmeceutical, and pharmaceutical industries. Additionally, the two-stage antioxidant behavior offers prospects for the formulation of straightforward, cost-effective analytical approaches to measure components in binary antioxidant formulations.

## 1. Introduction

Developing formulations that are both safe and effective by design is a concern for investigators who must address this issue in the design of each new product [1]. A number of environmental factors have a significant impact on a production unit during the manufacturing process and also after the product has left the factory. It is well established that oxidation is one of the processes with the potential to cause the loss of active components of a formulation, or even distort its activity [2,3,4,5,6].

Oxidation is a complex process involving a variety of mechanisms, sources, and targets of degradation [2]. There are a number of approaches that can be adopted in order to circumvent this phenomenon. These include the regulation of storage conditions, the preliminary elimination of oxidant impurities in excipients, specific packaging, and the addition of oxidant-protective supplements [2,7,8,9,10,11]. Antioxidants might be the solution of choice because their implementation rarely necessitates complex procedures or high costs. Moreover, quite often they work not only as excipients but also emerge with beneficial biological effects.

Flavonoids are among the most promising antioxidants, and many of them are being studied extensively due to their potential health benefits [12,13,14,15]. Among them, dihydroquercetin seems to be one of the most prospective for several reasons. First, it is a natural polyphenol with a high safety profile, favorable biopharmaceutical properties, and a wide range of biological activity [16,17,18,19,20,21,22,23]. Dihydroquercetin has been approved for use on the European Union market as a novel food for use in dietary supplements [24,25,26,27]. Another important point is that this flavonoid is produced on a high tonnage scale, so there is potential to meet the industrial needs of pharmaceutical, cosmeceutical, and food technologies. Market research data indicate that the global sales of dihydroquercetin will double from $60 million in 2025 to about $120 million in 2033 [28]. The versatility of its properties combined with a promising economic outlook makes it possible to develop novel formulations with enhanced therapeutic effects, such as anti-inflammatory or capillary-protective properties—in which dihydroquercetin will play a dual role as both active pharmaceutical ingredient and stabilizing excipient.

As an antioxidant, dihydroquercetin may be beneficial especially when combined with other radical-scavenging compounds since antioxidants often exhibit considerable mixture effects [29,30,31,32,33,34,35,36,37,38,39,40]. In particular, if the resulting antioxidant activity appeared to be higher than the anticipated additive sum, a synergy was considered, and, in the opposite case, an antagonism (subadditive effect) was postulated [29,41,42].

**Figure 1:**
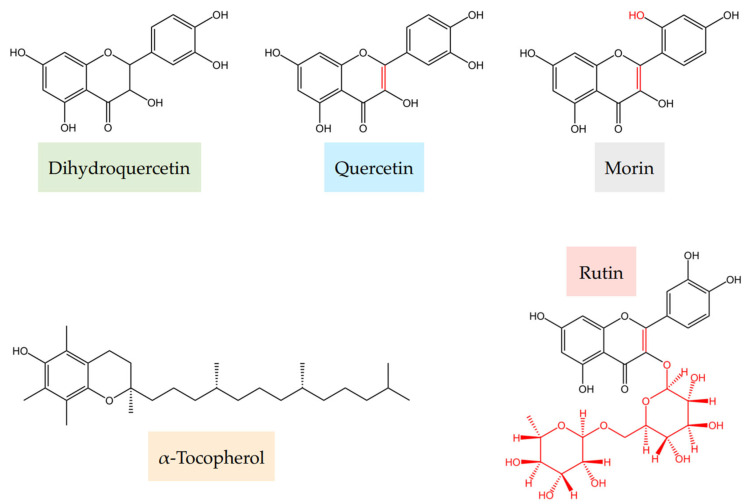
Structures of the components of the studied formulations. Differences are indicated by red markings.

Herein, we focused on formulations of dihydroquercetin with α-tocopherol. α-Tocopherol is a lipid-soluble endogenous antioxidant that exhibits high antioxidant activity. It is one of the most widely used antioxidants in the pharmaceutical, cosmeceutical, and food industries [42,43]. Together, flavonoids and α-tocopherol can potentially form an antioxidant system, covering both hydrophilic and lipophilic properties. Previously we found that formulations of dihydroquercetin with glutathione exhibit over 100% antioxidant synergy, and the mechanism is currently under investigation [31]. Thus, the systematic investigation of flavonoids with α-tocopherol antioxidant interactions, particularly with dihydroquercetin as a promising candidate, represents a step toward rational formulation design. We hypothesize that similarly to the individual activity of flavonoids, the effects of flavonoids in combination with other antioxidants are influenced by several structural fragments. As previously demonstrated, the most important fragments are the catechol type of B ring and the presence of a hydroxyl group in position 3 of ring C in conjunction with a C2-C3 double bond [44]. Together with dihydroquercetin, several structurally related flavonoids were also tested to provide insight into the structure–antioxidant mixture effect relationships, namely, quercetin as a dehydrogenated analog of dihydroquercetin with a longer chain of conjugation, rutin as a quercetin glucoside with blocked hydroxyl at the C-3 position, and morin as a flavonoid lacking a catechol group though able to form *p*-quinoid structure throughout the B and C rings of the flavonoids. All the studied structures are shown in Figure 1.

Since it was previously demonstrated that the antioxidant effect of formulations depends not only on the flavonoid structure and molar ratio but also on the mode of inflow of free radicals, we employed two different approaches based on 2,2′-azinobis(3-ethylbenzothiazoline-6-sulfonic acid) (ABTS) radical-cations scavenging activity: decolorization assay and lag-time assay [31].

## 2. Results

### 2.1. Antioxidant Capacity in the Decolorization Assay

#### 2.1.1. The ABTS^•+^ Radical-Cations Self-Bleaching

The decrease rate and absolute absorbance loss due to the self-bleaching of ABTS^•+^ radical-cations were maximal during the first 5–10 min, then declined steadily to reach generally negligible rates (see Table 1).

#### 2.1.2. Individual Compounds in the Decolorization Assay

**Figure 2:**
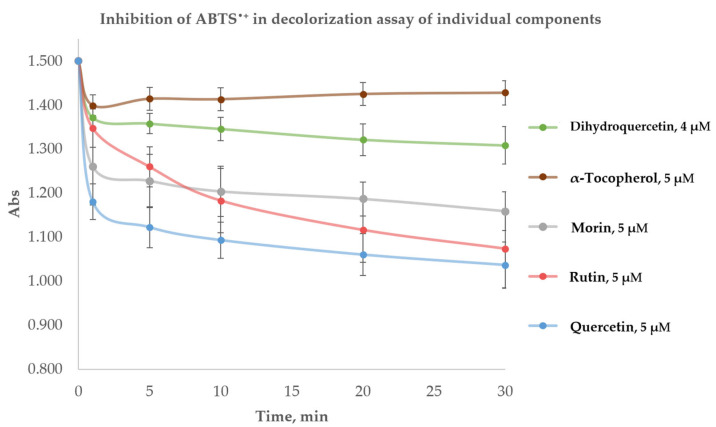
Averaged graphs of the two-step pattern of ABTS^•+^ inhibition by α-tocopherol, quercetin, morin, rutin at 5 μM and dihydroquercetin at 4 μM in decolorization assay.

A two-step decrease in absorbance was observed for all the flavonoids as shown in Figure 2. About 50–80% of the total ABTS^•+^ radical-cations were scavenged during the 1st minute. After the 1st minute, the absorbance decreased smoothly. α-tocopherol showed a different pattern of decrease in ABTS^•+^ radical-cations absorbance, inhibiting them totally during the 1st minute so that a stable plateau was observed throughout the rest of the incubation period. The total *n*-values from the 1st to the 30th min are shown in Table 2. Thus, the antioxidant with the greatest capacity appeared to be quercetin with an *n*-value equal to 6.34 ± 0.46 at 30 min, and α-tocopherol with an *n*-value of 1.45 ± 0.45 appeared to be the antioxidant with the lowest antioxidant capacity among those investigated.

#### 2.1.3. Flavonoid–α-Tocopherol Formulation at Different Molar Ratios

All formulations demonstrated additive or in some cases light subadditive interactions. No dependence on the concentration of the components was observed. Detailed data are given in Table 3.

### 2.2. Antioxidant Capacity in the Lag-Time Assay

#### 2.2.1. ABTS^•+^ Accumulation Without Antioxidants

**Figure 3:**
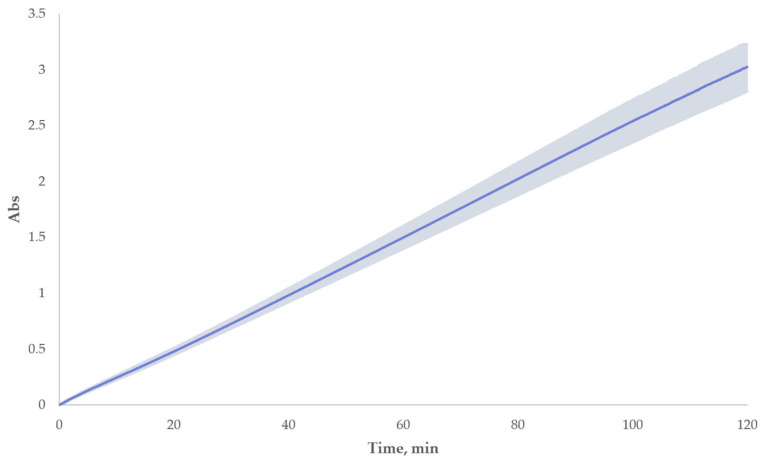
Averaged kinetic curve of ABTS^•+^ accumulation (dark blue) in ethanol without added antioxidant with standard deviation (light blue).

The ABTS^•+^ radical-cations accumulation without antioxidants reached an absorbance of 2.28 ± 0.16 after 90 min and had a linear tendency during at least the first 120 min of incubation, as can be seen from its kinetic curve in Figure 3. There were no significant changes in the ABTS^•+^ accumulation, particularly at the 1st, 30th, 60th, 90th, and 120th min; the rates were 0.025 ± 0.001, 0.024 ± 0.003, 0.023 ± 0.006, 0.026 ± 0.006, and 0.022 ± 0.022 respectively, the average rate was 0.024 ± 0.005. The coefficient of determination for the averaged curve was 0.9996.

#### 2.2.2. Individual Compounds in the Lag-Time Assay

The antioxidant capacity in terms of the *n*-values appeared to be 1.14 ± 0.07, 3.66 ± 0.53, 1.62 ± 0.07, 3.17 ± 0.13, and 1.22 ± 0.08 for dihydroquercetin, quercetin, morin, rutin, and α-tocopherol, respectively. The results obtained from individual runs were used in the mixture effect calculations for appropriate formulations.

**Figure 4:**
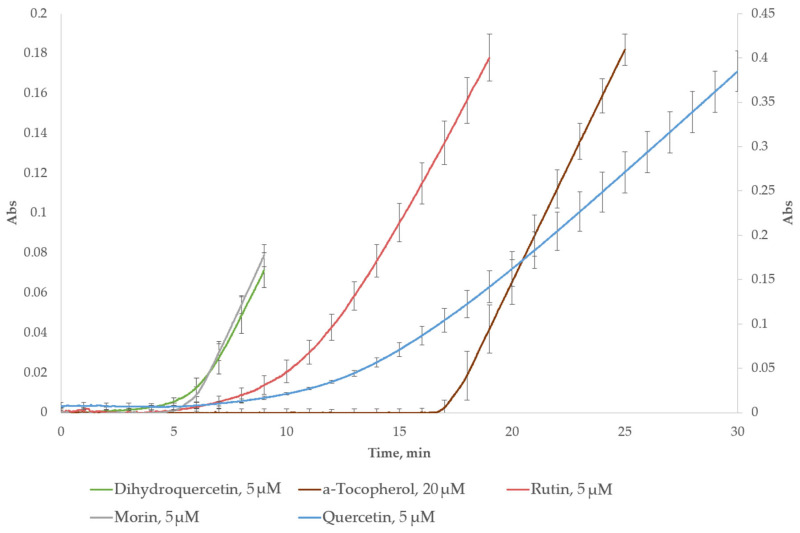
The kinetic curves of each of the compounds studied in their individual experiments. The quercetin curve is plotted on the secondary axis.

As illustrated in Figure 4, the kinetic curves for flavonoids consist of a period of complete inhibition of the accumulation of ABTS^•+^ radicals, followed by a gradual transition to an upward slope in the kinetic curve. For α-tocopherol, the lag phase in the kinetic curve ends abruptly, followed by an increase in the accumulation of ABTS^•+^.

#### 2.2.3. Flavonoid–α-Tocopherol Formulations at Different Molar Ratios

The examined formulations demonstrated mostly additive effects (see Table 4), except for quercetin–α-tocopherol 1:20, and dihydroquercetin–α-tocopherol 1:20, where only the minor deviations from the calculated sum of the individual components’ effects were observed.

#### 2.2.4. Lag-Time Complexity in Flavonoid–α-Tocopherol Formulations

**Figure 5:**
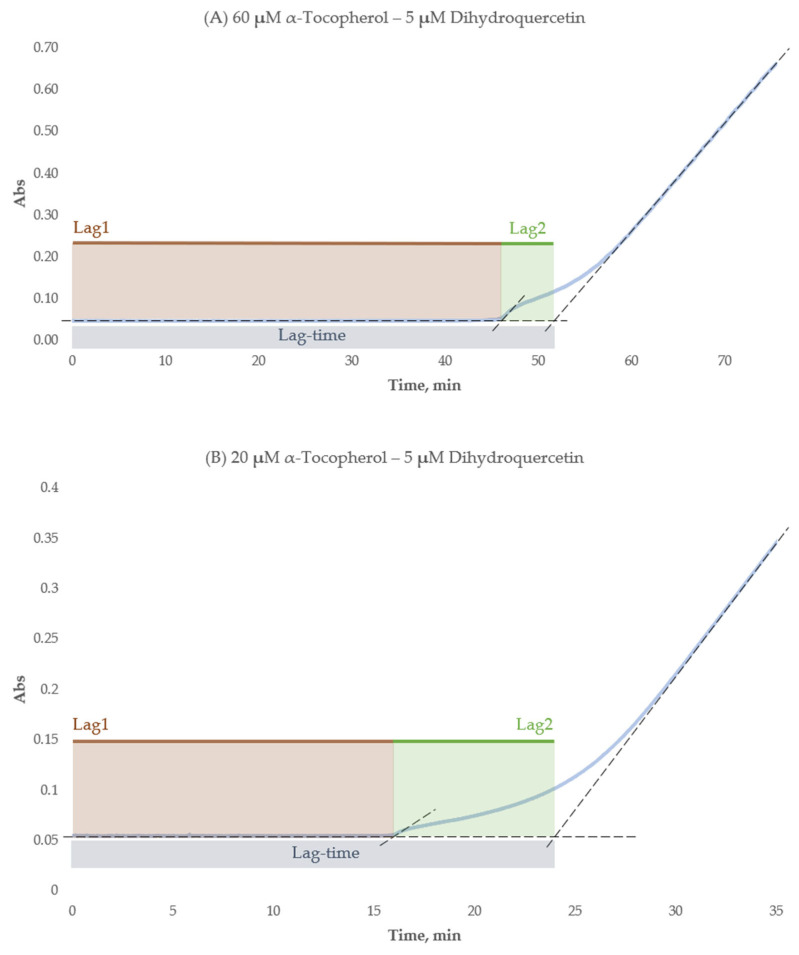
Graphical representation of three-staged lag-time experiment on different dihydroquercetin–α-tocopherol formulations. Light blue represents the kinetic curve, and the dashed lines show the tangent lines, which indicate the lag-time borders. (**A**) Formulation of 5 μM dihydroquercetin and 60 μM α-tocopherol, lag-time duration 52 min, Lag1—46 min, Lag2—6 min; (**B**) formulation of 5 μM dihydroquercetin and 20 μM α-tocopherol, lag-time duration 24 min, Lag1—16 min, Lag2—8 min.

A three-stage pattern was observed in the kinetic curves of all the formulations with two lag periods (Lag1 and Lag2), as shown in Figure 5. The initial stage (Lag1) manifested as an absorbance plateau, followed by a brief increase and then another plateau (Lag2). This latter stage transitioned smoothly into the third stage, characterized by steady absorbance growth until the end of the incubation period. Both Lag1 and Lag2 duration were measured by the tangent method as it was described previously [45,46].

**Figure 6:**
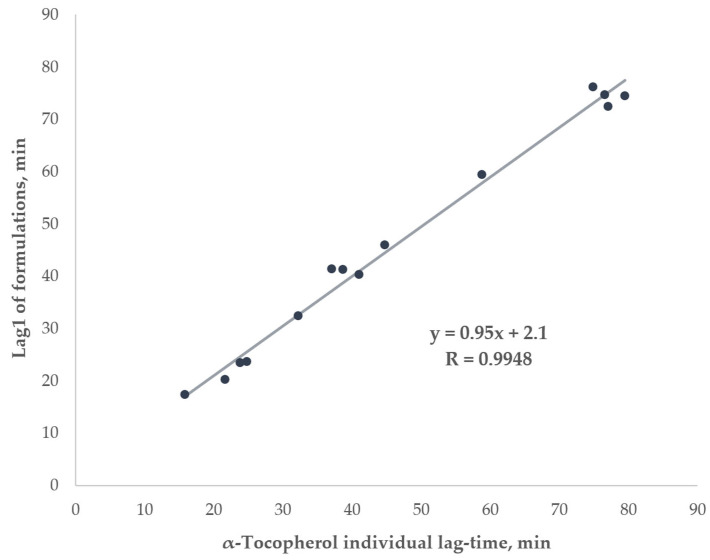
The strong correlation between the Lag1 in the examined formulations and the individual α-tocopherol lag-times at the same concentrations.

The Lag1 and Lag2 duration for formulations in comparison to the respective lag-times of their components in single experiments showed their strong similarity; the detailed information is given in Table 5. Lag1 durations closely matched α-tocopherol’s individual lag-times (correlation coefficient > 0.99), with negligible differences (see Figure 6). A similar observation was made for the individual lag-times of flavonoids and the corresponding Lag2 durations: out of the 14 formulations, in 8 cases the difference was insignificant; others were mostly negligible.

## 3. Discussion

### 3.1. Assessment of the Antioxidant Properties of Dihydroquercetin and Related Flavonoids with α-Tocopherol in Formulations

Antioxidant properties of formulations of α-tocopherol with different antioxidants have been studied previously. It was shown that α-tocopherol is conserved by water-soluble antioxidant ascorbic acid in a homogeneous alcoholic environment so that its level in the reaction mixture remains almost the same until the consumption of ascorbic is complete [47]. One of the possible mechanisms of such interaction could be the reduction in one antioxidant by another, the so-called sacrificial antioxidant. The antioxidant behavior of flavonoid–α-tocopherol formulations was examined in several studies and demonstrated to vary in different conditions. For example, in human blood cell membranes, quercetin and α-tocopherol inhibited the photooxidation of each other [48]. Electrochemically generated radicals of quercetin, dihydroquercetin, catechin, and luteolin were efficiently regenerated by α-tocopherol in a homogeneous solution of dimethylformamide [49]. Multi-component formulation with α-tocopherol, quercetin, and several other antioxidants showed subadditive interaction in azo-initiated peroxidation of lipids in liposomes [50]. Another study on the antioxidant effect of α-tocopherol in the presence of green tea polyphenols in SDS micelles revealed the regeneration of α-tocopherol with a noticeable synergy [32,51]. A particularly noteworthy finding was that quercetin exhibited the strongest synergistic effect with α-tocopherol at a ratio of 2:1 in the olive oil model. Furthermore, the analysis of the dynamic changes of antioxidants during oxidation indicated that the repair and regeneration of α-tocopherol by quercetin were responsible for the revealed synergistic effect [52]. In this study, we investigated formulations containing dihydroquercetin and structurally analogous flavonoids with α-tocopherol in simple oxidation models with a wide range of ratios to clarify the mixture effect in homogenous polar solvents. Initially, we suggested that flavonoids might be sacrificial antioxidants for α-tocopherol, thus different flavonoids were tested at different concentrations to see if some formulations would produce the desired synergism.

To assess the interaction between the antioxidants, we employed two simple oxidation models, the decolorization assay and lag-time assay. Both models are based on the inhibition of the same model radical but in different oxidation conditions. Briefly, the decolorization assay is the experimental design with a high ABTS^•+^:antioxidant ratio, sometimes exceeding 20:1 [53]. Therefore, the ABTS^•+^ radical is in great excess so that the reaction goes deeper with higher *n*-values on individual antioxidants [31,45]. On the other hand, the molecules of antioxidants meet radical-cation more than another antioxidant molecule, so the effective antioxidant network is scarcely formed. The lag-time assay is profoundly different since the concentration of ABTS^•+^ during a lag-time period is close to zero as the molecules of antioxidants scavenge ABTS^•+^ as they appear in situ, keeping them from accumulating. That allows the antioxidants to cooperate leading to the formation of reactive adducts or the reduction of one antioxidant by another. This cooperation could lead to a change of the lag-time of formulation in comparison with the simple sum of its components revealing a synergistic or antagonistic effect.

Antioxidant assays yield conflicting data due to inherent chemical biases, non-standardized conditions, and divergent biological relevance [54,55,56]. Despite the absence of a universally applicable assay, the integration of diverse methodologies or the prioritization of mechanism-driven interpretations can mitigate the occurrence of misinterpretations. It is imperative that the field evolves from the utilization of ‘total antioxidant capacity’ as a standalone metric towards incorporating additional kinetic profiling, in vivo validation, etc. [57,58,59]. In the present study, rather than combining several divergent antioxidant assays, which frequently yield unrelated and even controversial data that is challenging to interpret, the focus was directed towards two distinct 2,2′-azino-bis[3-ethylbenzothiazoline-6-sulfonic acid] radical radical–cation scavenging approaches, grounded in decolorization lag-time or end-point experimental strategies [45,60]. The former demonstrated greater promise in combination effect studies. Conversely, the latter study exhibited reduced sensitivity to manifestations of mixture effects; nevertheless, it remained adequate in estimating the total antioxidant capacity of complex combinations.

In agreement with the previously reported studies, α-tocopherol demonstrated its “fast-reacting” properties in the decolorization assay, revealing its total activity during the 1st min, while flavonoids kept inhibiting ABTS^•+^ much longer [61,62,63,64,65,66,67,68,69,70,71,72]. At the same time, the antioxidant capacity of flavonoids was several times higher than that of α-tocopherol. Such formulations may combine the advantages of rapid radical scavenging of α-tocopherol and the prolonged oxidation elimination of flavonoids. Nevertheless, we failed to detect any significant mixture effects in the decolorization assay (Table 3).

In the lag-time assay, both individual antioxidant capacities of individual flavonoids and α-tocopherol as well as their formulations were in agreement with the decolorization assay results. It seems that, due to its inherent properties, α-tocopherol reacts readily with ABTS^•+^ with no formation of intermediates or products that enter in any substantial interaction with flavonoids to gain synergy and antagonism. Also, that means that the presence of both α-tocopherol and flavonoids in the water–alcohol medium does not result in the formation of more active conjugates that could enhance the mixture’s overall antioxidant activity through synergy. Previously similar additive mixture effects were revealed for flavonoids with α-tocopherol. For example, M.N. Peyrat-Maillard et al. carried out a study on the antioxidant effects of various polyphenolic compounds with α-tocopherol and also obtained additive effects for the quercetin–α-tocopherol formulation [72]. They estimated oxidation in the gradual radical inflow mode, indicating the interval of inhibition of radical accumulation, as we have done in the current work, though the experimental design was different and 2,2′-azobis (2-amidinopropane) dihydrochloride was used to initiate oxidation in aqueous dispersion of linoleic acid. However, one phenomenon we noticed encouraged us to go into detail.

In the lag-time assay, the individual antioxidants and their formulations had a pronounced lag-time in their kinetic curves. Typically, a kinetic curve can be broken into two stages. This pattern was reported by different researchers in similar ABTS^•+^ radical-cationbased lag-time studies and includes a plateau period, when the main antioxidant property is manifested, and the subsequent increase in absorbance, when the reaction mixture is running out of antioxidant and the accumulation of ABTS^•+^ becomes steady [73,74,75,76].

Interestingly, we observed the three-staged pattern for α-tocopherol with flavonoid formulations in our experiments as it was illustrated in Figure 3. The first stage was the same as before—the plateau took place from the beginning, but the increase in absorbance appeared twice—as the sharp increase and subsequent smooth increase. Such a pattern allowed us to assume that the antioxidants react one after another. After breaking down the lag-time of the formulations into two lags (Lag1 and Lag2, see Figure 5), we observed some similarities in the duration of the latter compared to the corresponding lag-times of the individual compounds. This can be seen in detail in Table 5, particularly, the difference between Lag1 of the formulations (Table 5, column 4) and the corresponding lag-time of α-tocopherol in single experiments at the same concentration (Table 5, column 6) was mostly insignificant, yielding a correlation coefficient close to 1 (see Figure 6). Similar analysis of Lag2 of the formulations in comparison with the corresponding lag-times of individual flavonoids also showed a considerable resemblance between them (Table 5, columns 5 and 7). All in all, we assumed that in all formulations during Lag1, mainly α-tocopherol exerts its antioxidant effect, while Lag2 is the period when flavonoid participates in the radical scavenging. That allowed us to suggest that α-tocopherol can serve as a protective agent for dihydroquercetin and related flavonoids against radical oxidation. Even though α-tocopherol has a lower antioxidant capacity than the flavonoids, its rate of radical-scavenging could be expected to be higher. In particular, it has been shown that the constant rate of peroxyl radicals inhibition for α-tocopherol is at least 5–10-fold higher in organic solvents in comparison to flavonoids [77,78,79]. However, in polar solvents this situation is not that unambiguous; for example, the antioxidant performance of quercetin in peroxyl radicals trapping at physiologically mimetic pH 7.4 approached that of 2,2,5,7,8-pentamethyl-6-chromanol, a very close analog of α-tocopherol (vitamin E), apparently due to the sequential proton-loss electron transfer, SPLET (the sequential proton loss electron transfer) mechanism [79,80]. Our data suggests that in the water–alcohol media, α-tocopherol still dominates despite being a classic lipophilic radical-trapping antioxidant. This finding holds potential for industrial applications in the domains of nutraceuticals, pharmaceuticals, and cosmetics.

Additionally, as the revealed two-phase lag-time pattern in the case of the examined formulation appeared to be quantitatively reproduced, there is some potential to use it in the quantitative determination of components, considering that the lag-time assay is a simple, undemanding, and cost-effective approach. Recently, Francisco R. Marin et al. described the development of a new, rapid analytical approach to assess flavonoids. This approach relied on the peroxidase-mediated interaction of ABTS with polyphenols, and it involved the spectrophotometric determination of the ABTS-flavonoid adducts formed [76]. In our study, we identified differences in lag-times for certain formulations. Consequently, further studies are being performed to adjust it for the quantitative determination of dihydroquercetin-based formulations.

### 3.2. Methodological Considerations

A few methodological considerations need to be regarded in terms of utilizing the decolorization and lag-time assays.

Previously it was reported that the self-bleaching of ABTS^•+^ needs to be accounted for in decolorization assay experiments [31,65,81]. This was even more important in the case of our experiments as the solvent was of an alcoholic nature, and it appeared that the self-bleaching was strongly dependent on the quality of the solvent. We examined the phenomenon of self-bleaching, comparing two distinct samples of ethanol, and found a substantial variation in the rate of ABTS^•+^ absorption loss. While the ABTS^•+^ bleached almost negligibly with the fresh ethanol losing only about 0.04 ± 0.004 absorbance units from the initial 1.5 by the 30th min, the approximately one-year-old ethanol showed a considerable absorbance loss of 0.4 ± 0.05 absorbance units. We suppose that the reason is a peroxide formation. While generally ethanol is not prone to peroxide formation as isopropanol or ethers are [82,83], they possibly may accumulate in sufficient concentrations when ethanol is used as a solvent. Even if only a small part of ethanol transforms into peroxides, their total amount may become significant enough to interact with radical-cations and influence the results. To ensure consistency, we exclusively used fresh ethanol in our measurements.

In the case of a lag-time assay, the approach for a lag-time estimation can sometimes be tricky. As the rate of ABTS^•+^ accumulation differs between the first 5 min and, for instance, 1 h, we need to account for this in our estimation of antioxidant properties. That is why we examined ABTS^•+^ accumulation characteristics before the primary experiments. In our previous study, which was performed in PBS, we observed that the accumulation rate differed throughout a single measurement [31]. However, the accumulation of radical-cations in ethanolic solution appeared to change negligibly during the first 120 min of the experiment which was enough for all our samples. So, we could use lag-time duration as a comparative parameter without any additional calculations. At the same time, there was another source of error, which was the tangent approach used to determine the duration of a lag-time. If there was a sharp transition from the plateau part of a kinetic curve to the rising part, a lag-time could be measured directly. However, if the transition was smooth, the tangent approach was used. The most difficulties were encountered when measuring Lag2, as it was much shorter than Lag1 and had a small share in the total duration of the lag-time of a formulation. For example, in the case of the formulations studied, Lag2 decreased as the α-tocopherol concentration increased, while the flavonoid concentration remained constant. Thus, it is difficult to say whether the reason was an ambiguous determination of Lag2 or whether there was another cause, such as the possible reaction of a flavonoid component with intermediates and/or oxidation products instead of ABTS^•+^ inhibition, or a partial contribution of a flavonoid component to Lag1 that was not captured due to its relative negligibility with the simultaneous shrinking of Lag2.

## 4. Materials and Methods

### 4.1. Materials

#### 4.1.1. Components of Flavonoid–α-Tocopherol Formulations

Dihydroquercetin was purchased from JSC Ametis (Blagoveshchensk, Russia); quercetin, rutin, morin, α-tocopherol, purum ethanol (>99.8%), and *N*,*N*-dimethylformamide (99%) were sourced from Acros Organics (Geel, Belgium).

#### 4.1.2. Components of ABTS/PP Oxidizing System

2,2′-Azinobis(3-ethylbenzothiazoline-6-sulfonic acid) diammonium salt (ABTS) and potassium persulfate (di-potassium peroxydisulfate, PP) were obtained from Sigma-Aldrich (Burlington, MA, USA).

### 4.2. Preparation of Solutions

Ethanol (≥99.8%) from Sigma-Aldrich (Burlington, MA, USA), deionized 18 MΩ·cm water from LLC Smoly (Moscow, Russia), and *N*,*N*-dimethylformamide (99%) from Acros Organics (Geel, Belgium) were used for the preparation of solutions.

Stock solutions of dihydroquercetin, quercetin, morin, and α-tocopherol were prepared in ethanol, whereas rutin was dissolved in 1:10 *N*,*N*-dimethylformamide-ethanol mixture, PP in DI (deionized) water. All the formulations were prepared in ethanol. For the decolorization assay, ABTS was dissolved in DI water; for the lag-time assay, ABTS was dissolved in a small amount of DI water and then diluted in ethanol.

None of the solvents interfered with the assays. The solutions were prepared on the same day as they were used.

### 4.3. Methods

All manipulations were carried out using spectrophotometric analysis by Cary 100 (Varian, Palo Alto, CA, USA) with a 1 cm Peltier temperature-controlled cuvette with a path length of 1 cm.

#### 4.3.1. Decolorization Assay

**General procedure.** The decolorization assay was performed as reported by Re et al. with minor modifications [60]. The antioxidant sample was added to the stock ABTS^•+^ solution and placed in a heating chamber at 30 °C for 30 min after shaking for 15 s. The decrease in absorbance was read spectrophotometrically at 750 nm maxima on the 1st min, 5th min, 10th min, 20th min, and 30th min with a preliminary measurement of the initial absorbance (A_initial_) in the absence of the antioxidant sample. A_initial_ was 1.5 ± 0.1 for all measurements.

**Generation of ABTS^•+^.** Aqueous solutions of ABTS and PP were prepared in concentrations of 7 mmol/L and 2.5 mmol/L, respectively. These solutions were mixed in a volume ratio of 1:1 and kept in the dark at room temperature for 10 h to obtain the stock ABTS^•+^ solution which was exploited during the next 1–2 days.

**ABTS^•+^ self-bleaching.** ABTS^•+^ self-bleaching measuring involves the same steps as the general procedure but with the addition of ethanol 10 μL instead of an antioxidant sample.

***n*-Value and mixture effect calculations.** The ABTS^•+^ absorbance decrease (∆A) was calculated as follows:

∆A = A_initial_ − A_x_ + ∆A^self-bleaching^, (1)

where A_initial_ and A_x_ are the ABTS^•+^ absorbances at 750 nm, immediately before the addition of the antioxidant sample (zero time point) and after a certain incubation time (x min) with the sample, respectively. The ABTS^•+^ self-bleaching (∆A^self-bleaching^) is the difference between the ABTS^•+^ absorbance at a zero time point and after a corresponding time of incubation (x min) with the addition of ethanol instead of the antioxidant sample.

The amount of ABTS^•+^ radicals scavenged by one molecule of antioxidant (*n*-value) was calculated using the following equation:

*n*-value = C^ABTS•+^/C^antioxidant^ = ∆A/(ε × C^antioxidant^ × l), (2)

where C^ABTS•+^ is the concentration of scavenged ABTS^•+^, C^antioxidant^ is the concentration of the antioxidant sample, ε is the extinction coefficient equal to 15,000 L·mol^−1^·cm^−1^, and l is the optical pathlength of 1 cm.

The decolorization mixture effect (ME) has been calculated in the following way:

ME_Decolorization_ = ((∆A^mix^_experimental_ − ∆A^mix^_theoretical_)/∆A^mix^_theoretical_) × 100%, (3)

where ∆A^mix^_experimental_ and ∆A^mix^_theoretical_ were calculated following the next equation:

∆A^mix^_experimental_ = A^mix^_initial_ − A^mix^_30_ + ∆A^self-bleaching^_30_, (4)

∆A^mix^_theoretical_ = A^mix^_initial_ − (∆A^1^_30_ + ∆A^2^_30_) + ∆A^self-bleaching^_30_, (5)

where ∆A^1^_30_ and ∆A^2^_30_ are the decrease in absorbance at the 30th min of component 1 and component 2, respectively, A^mix^_initial_ is the initial absorbance of these components mixed.

#### 4.3.2. Lag-Time Assay

**General procedure.** The lag-time assay was performed as reported by Ilyasov et al. [31,45]. Briefly, an antioxidant sample is added to the ABTS solution before the PP. Absorbance was monitored continuously at a wavelength of 750 nm with a frequency of 0.5 s^−1^.

The antioxidant sample was mixed with 3.3 mL of ABTS solution in a volume of 10–40 μL. Then, 0.5 mL of PP solution was added and the mixture was thoroughly shaken for 15 s. The final concentrations of ABTS and PP in the solution are 4.5 mM and 1.5 mM, respectively. Antioxidant concentrations were selected to ensure a minimum lag-time of 3 min, thereby reducing preparation-related errors.

**Calculation of *n*-value and mixture effect.** The amount of ABTS^•+^ radicals scavenged per one molecule of antioxidant (*n*-value) in the lag-time assay was calculated as follows:

*n*-value = C^expected ABTS•+^/C^antioxidant^, (6)

where C^expected ABTS•+^ is the concentration of ABTS^•+^ that would accumulate by the end of lag-time without the addition of antioxidants, and C^antioxidant^ is the concentration of antioxidant.

The expected accumulated ABTS^•+^ concentration was calculated by dividing the absorbance A_lag-time_ at a time point corresponding to a certain lag-time by an extinction coefficient ε of 15,000 L×mol^−1^×cm^−1^ for the ABTS^•+^ radical cation and a path length l of 1 cm.

C^expected ABTS•+^ = A_lag-time_/(ε × l), (7)

The corresponding absorbance value at each time point A_lag-time_ was derived from the mean of several independent kinetic curve runs at 750 nm.

The lag-time mixture effect was calculated according to the following equation:

ME_lag-time_ = ((lag-time_experimental_ − lag-time_theoretical_)/lag-time_theoretical_) × 100%, (8)

where lag-time_experimental_ is the empirically obtained lag-time duration, of the studied formulation, in min; lag-time_theoretical_ is the calculated sum of the individual components’ lag-times for the studied formulation, in min.

### 4.4. Statistics

All data are presented as mean ± standard deviation (SD) from at least three independent determinations. Experimental and simulation results were analyzed using the paired Student’s *t*-test.

## 5. Conclusions

Our study allows us to assume that the examined flavonoids remain mostly intact while α-tocopherol is present in the reaction mixture in the alcoholic medium. This suggests that they are protected from oxidation in these formulations, but further investigations are needed. This can be important in the industries that utilize, research, and develop flavonoids such as the nutraceutical, cosmeceutical, and pharmaceutical industries. In addition, the two-phase lag pattern offers the prospect of developing simple and cost-effective analytical quantitative determination approaches for binary formulations after appropriate customization and validation.

## Figures and Tables

**Table 1:** The self-bleaching of the ABTS^•+^ radical-cations without the addition of antioxidants at initial absorbance of 1.5 (120 µM, 750 nm).

	Time Interval, min			
	0–5	5–10	10–20	20–30
Decrease rate, µM × min^−1^	0.10 ± 0.03	0.11 ± 0.00	0.06 ± 0.01	0.05 ± 0.01
Absolute absorbance loss, a.u. × min^−1^	0.015 ± 0.002	0.008 ± 0.001	0.008 ± 0.002	0.008 ± 0.002

**Table 2:** The *n*-values for flavonoids and α-tocopherol at different incubation times. All *n*-values were statistically different (*p* < 0.05).

Component	*n*-Value SD (% of the Total *n*-Value *)				
	1 min	5 min	10 min	20 min	30 min
Dihydroquercetin	2.14 ± 0.20 (76 ± 7)	2.25 ± 0.19 (80 ± 7)	2.40 ± 0.22 (85 ± 8)	2.67 ± 0.31 (95 ± 11)	2.82 ± 0.41
Quercetin	4.21 ± 0.44 (66 ± 3)	5.10 ± 0.20 (81 ± 3)	5.78 ± 0.32 (91 ± 8)	6.48 ± 0.68 (102 ± 6)	6.34 ± 0.46
Rutin	2.09 ± 0.39 (47 ± 12)	2.99 ± 1.02 (64 ± 7)	3.73 ± 1.44 (78 ± 7)	4.37 ± 1.70 (91 ± 7)	4.73 ± 1.60
Morin	3.78 ± 0.91 (74 ± 11)	4.16 ± 0.71 (82 ± 5)	4.56 ± 0.74 (89 ± 5)	4.73 ± 0.64 (93 ± 2)	5.08 ± 0.60
α-Tocopherol	1.52 ± 0.17 (110 ± 23)	1.44 ± 0.25 (102 ± 14)	1.43 ± 0.22 (102 ± 16)	1.36 ± 0.27 (96 ± 11)	1.45 ± 0.45

* The *n*-value after 30 min incubation was taken as 100%.

**Table 3:** Antioxidant mixture effects of the flavonoid–α-tocopherol formulations in the decolorization assay.

Formulation	Ratio	Concentrations, μM–μM	Decolorization Mixture Effect, %
Dihydroquercetin–α-Tocopherol	1:1	12–12	−13.0 ± 2.9 ^b^
	1:5	4–20	−11.1 ± 2.7 ^b^
	1:10	4–40	−9.1 ± 3.2 ^b^
Quercetin–α-Tocopherol	1:1	5–5	−8.9 ± 5.0 ^a^
	1:5	2.5–12.5	−7.1 ± 3.6 ^a^
	1:10	2.5–25	−8.0 ± 1.5 ^b^
Rutin–α-Tocopherol	1:1	5–5	−10.3 ± 3.1 ^b^
	1:5	2.5–12.5	−4.4 ± 1.4 ^b^
	1:10	2.5–25	−3.2 ± 3.3 ^a^
Morin–α-Tocopherol	1:1	7.5–7.5	−9.4 ± 3.7 ^b^
	1:5	5–25	−9.5 ± 5.8 ^a^
	1:10	2.5–25	−12.5 ± 1.4 ^b^

^a^—statistically insignificant, *p* > 0.05; ^b^—statistically significant with *p*-value less than 0.05.

**Table 4:** Mixture effects of flavonoid–α-tocopherol formulations in the lag-time assay.

Formulation	Ratio	Concentration, µM–μM	Lag-Time Mixture Effect, %
Dihydroquercetin–α-Tocopherol	1:4	5–20	1.2 ± 3.8 ^a^
	1:8	5–40	3.6 ± 3.6 ^a^
	1:12	5–60	2.3 ± 4.4 ^a^
	1:16	5–80	2.7 ± 5.9 ^a^
	1:20	5–100	7.2 ± 1.7 ^b^
Quercetin–α-Tocopherol	1:5	5–25	−1.7 ± 7.5 ^a^
	1:10	5–50	−14.4 ± 15.2 ^a^
	1:20	5–100	−12.9 ± 2.0 ^b^
Rutin–α-Tocopherol	1:5	5–25	−2.3 ± 7.2 ^a^
	1:10	5–50	−9.3 ± 2.2 ^b^
	1:20	5–100	−6.1 ± 3.9 ^a^
Morin–α-Tocopherol	1:5	5–25	0.6 ± 2.2 ^a^
	1:10	5–50	1.0 ± 13.9 ^a^
	1:20	5–100	4.6 ± 6.5 ^a^

^a^—statistically insignificant, *p* > 0.05; ^b^—statistically significant with *p*-value less than 0.05.

**Table 5:** The durations of the Lag1 and Lag2 in flavonoid–α-tocopherol formulations in comparison to the lag-time duration of their individual components.

Formulation	Ratio	Concentration, µM	Duration of Lags in Lag-Time of Flavonoid–α-Tocopherol Formulation, min		Individual Lag-Time of Component with Concentration Corresponding to Formulation, min	
			Lag1	Lag2	α-Toc	Flavonoid
1	2	3	4	5	6	7
Dihydroquercetin – α-Tocopherol	1:4	5–20	15.8 ± 0.2 ^b^	7.4 ± 0.9 ^a^	17.2 ± 0.6	5.7 ± 0.2
	1:8	5–40	32.2 ± 0.3 ^a^	6.8 ± 0.4 ^b^	32.4 ± 1.3	
	1:12	5–60	44.8 ± 3.2 ^a^	7.3 ± 2.1 ^a^	46.0 ± 2.8	
	1:16	5–80	58.8 ± 3.7 ^a^	6.6 ± 0.9 ^a^	59.4 ± 3.9	
	1:20	5–100	77.0 ± 1.2 ^b^	6.2 ± 0.5 ^a^	72.3 ± 2.1	
Quercetin – α-Tocopherol	1:5	5–25	21.7 ± 1.4 ^b^	10.6 ± 0.9 ^b^	20.2 ± 0.7	13.3 ± 2.0
	1:10	5–50	37.2 ± 1.4 ^a^	9.2 ± 3.7 ^a^	41.3 ± 1.8	
	1:20	5–100	74.9 ± 3.3 ^a^	8.8 ± 0.3 ^b^	76.0 ± 0.1	
Rutin – α-Tocopherol	1:5	5–25	24.9 ± 1.2 ^a^	8.2 ± 1.2 ^b^	23.6 ± 1.5	11.5 ± 0.5
	1:10	5–50	38.8 ± 2.4 ^b^	9.1 ± 0.9 ^b^	41.1 ± 3.1	
	1:20	5–100	76.7 ± 2.2 ^a^	8.6 ± 3.4 ^b^	74.7 ± 5.5	
Morin – α-Tocopherol	1:5	5–25	23.5 ± 2.3 ^a^	6.0 ± 0.3 ^b^	23.4 ± 1.9	5.5 ± 0.2
	1:10	5–50	41.0 ± 4.2 ^a^	4.8 ± 0.5 ^b^	40.3 ± 3.6	
	1:20	5–100	78.8 ± 4.0 ^a^	4.8 ± 1.2 ^a^	74.4 ± 5.9	

^a^—statistically insignificant, *p* > 0.05; ^b^—statistically significant with *p*-value less than 0.05.

## Data Availability

The data supporting the findings of this study are available within the article and upon request.
